# Factors associated with self-medication of antibiotics by caregivers in pediatric patients attending the emergency department: a case-control study

**DOI:** 10.1186/s12887-022-03572-z

**Published:** 2022-09-01

**Authors:** Jhon Camacho Cruz, Carolina Zambrano Perez, Maria Carolina Sánchez Cabrera, Estefania Robledo Lopez, Pablo Vásquez Hoyos, Diana Rojas Rojas, Andrea Ortiz Montaña

**Affiliations:** 1 Fundación Universitaria de Ciencias de la Salud (FUCS), Bogotá, Colombia; 2 Department of Pediatrics, Sociedad de Cirugía de Bogotá - Hospital de San José, Calle 10 No.18-75, Bogotá, Colombia; 3 Department of Pediatrics, Hospital Infantil Universitario de San José, Bogotá, Colombia

**Keywords:** Anti- bacterial agents, Self-medication, Emergency Medical Services, Caregivers

## Abstract

**Introduction:**

Antibiotic self-medication is a common practice in pediatric caregivers in low-income countries with limited resources and represents a public health problem. Our study sought to determine what factors are associated with this practice, including differences in knowledge or attitudes of caregivers who attend a pediatric emergency service.

**Methods:**

Case-control study based on surveys of caregivers of pediatric patients brought to the emergency room with clinical symptoms suggestive of acute infection. Cases were defined as those caregivers who reported self-medication of antibiotics for the current illness and controls where those who did not report self-medication. Information was collected through a self-administered questionnaire that inquired about demographic and family characteristics, attitudes and knowledge toward self-medication of antibiotics. Data were compared using logistic regression and are presented with odd ratios and confidence intervals.

**Results:**

A total of 728 caregivers, 182 cases and 546 controls were included. We found that higher parental education, both in mothers (OR 0.56, 95% CI 0.40-0.79) and fathers (OR 0.62, 95% CI 0.43-0.89) was associated with less self-medication. Attitudes such as always requesting antibiotics from their doctors (OR 3.92, 95% CI 1.59-9.66), frequently buying antibiotics without a prescription (OR 23.66, 95% CI 11.76-47.59) and giving advice on antibiotics among family members (OR 2.90, 95% CI 1.75-4.82) resulted in an increased likelihood of self-medication. There was also a higher probability of antibiotic self-medication in older children (OR 1.13, 95% CI 1.09-1.17), those with a greater number of siblings (OR 1.25, 95% CI 1.09-1.43) and in those cases that received antibiotics within the last 3 months (OR 6.27, 95% CI 4.35-9.04). Overall knowledge of risk of antibiotic self-medication was low.

**Conclusions:**

Some patient and family characteristics such as age, number of siblings, recent antibiotic usage and inappropriate attitudes are strongly related to antibiotic self-medication. These findings will inform future interventions to reduce self-medication in children.

## Introduction

Self-medication is defined as the use of medication to treat self-diagnosed symptoms, or the intermittent or continued use of a prescribed drug for chronic or recurrent disease [1]. Antibiotics are one of the most widely used medications in both developed and developing countries, but are often misused, especially in low-resource settings [2]. Lack of awareness of doses, treatment schemes and other medication characteristics increases the risk of adverse effects and allergic reactions. Self-medication may also mask a more serious disease due to partial treatment, and may increase morbidity and mortality of infections due to development of multidrug-resistant bacteria [3]. This practice is especially dangerous in pediatric patients due to the lack of controlled clinical trials evaluating the efficacy and safety of a wide range of medications [4]. Furthermore, the vast majority of self-medicated antibiotics are used to control viral upper respiratory tract infections, which are self-limited illnesses that do not respond to antibiotic therapy [3, 5]. The phenomenon of self-medication is facilitated by the free access to antibiotics without a prescription in pharmacies, the lack of knowledge of caregivers about the indication and existence of different types of antibiotics, the pressure on doctors to prescribe them and the high frequency of multidrug-resistant infections [6, 7].

To our knowledge, there are no studies in the pediatric population that explore factors associated with antibiotic self-medication in the emergency room. We believe that this is a particularly vulnerable population that needs more research. That is why our study seeks to determine what factors are associated with antibiotic self-medication by caregivers, including differences in knowledge or attitudes, in pediatric patients with symptoms of infection that are taken to the emergency room.

## Materials and methods

### Study design and patient selection

We chose a concurrent case-control study model, as studies in adults reported a low frequency of self-medication (7%) [8]. The study was performed between July 2019 to July 2020 using a questionnaire completed by caregivers Pediatric patient caregivers were recruited in the pediatric emergency rooms of two general teaching hospitals that provide care under an insured care model and cover a middle-income population in the city of Bogotá, Colombia. This study was reviewed and approved by the local ethics committee (IRB00011307; 0271-2019). An informed consent was obtained from all subjects. Only patients older than 1 month and younger than 18 years, who were determined to have an acute infection at the end of the initial medical evaluation were selected for screening. Cases were selected if the treating physician had diagnosed an acute infection and the caregiver reported self-medication, defined as the administration of an antibiotic to the child during the current infection that was not prescribed by a physician. Cases of self-medication with topical antibiotics were not included. Three controls were then chosen immediately after each event from patients with an acute infection whose caregivers did not report self-medication.

### Questionnaire design

The questionnaire, presented in Spanish, was developed by the investigators to explore the factors related to antibiotic self-medication. First, a bibliographic search was carried out to establish which variables could be associated, such as personal or family factors, and knowledge and attitudes of caregivers towards self-medication with antibiotics. Based on the information extracted [9–16] a standardized data collection questionnaire was designed for caregivers to complete independently, but under the supervision of one of the researchers. Initially, the questionnaire inquired about some demographic characteristics of the patient (age, sex, weight and height, medical history, previous use of antibiotics in the last 3 months, number and position among siblings, history of siblings who received antibiotics in the last 3 months), and caregivers (relationship with the patient, socioeconomic level, area of ​​residence, type of insurance, education of the patient's parents). Then, the following questions focused on characterizing the attitudes of caregivers towards self-medication (storing of antibiotics at home, use of antibiotics for fever, asking for antibiotics without a prescription) and finally questions on knowledge of caregivers on the risks of self-medication. (adverse effects, resistance to antibiotics, use of antibiotics for viral infections). All questions were closed questions. To establish face and content validity, the questionnaire was piloted with 15 caregivers, and their suggestions were incorporated. An introductory paragraph explaining the main objective of the study was added.

### Study procedure

The research team approached all caregivers after the physician had completed the initial medical assessment and after informed consent was obtained from the caregiver. Caregivers were given an electronic tablet to complete the self-administered questionnaire under the researchers’ supervision. If the caregiver had difficulty reading or was illiterate, a person independent of the study was assigned to read the questions and help write the responses.

All caregiver's queries were resolved during questionnaire completion in the emergency room to minimize errors and missing data. Recruitment was done 24 hours a day by the physician on call and each new case was supervised and reviewed by a member of the investigation team.

### Statistical analysis

The sample size was calculated expecting a frequency of exposure for the control group to a study factor equal to or greater than 8%, an OR greater than 2, with a power of 80% and an alpha error of 0.05, which determined the need for 210 cases and 630 controls. Due to the COVID-19 pandemic, recruitment was interrupted, so the power was recalculated for the sample size obtained (182 cases and 546 controls) and it turned out to be 75%, so the research group considered it acceptable for analysis. A descriptive analysis of cases and controls was first performed separately. Based on the distribution of the variables, categorical variables were expressed in absolute numbers and percentages and continuous variables as medians and interquartile ranges (IQR) (since all the continuous variables studied followed a left-skewed distribution). For missing data, we decided to omit these cases and analyze the remaining data (list-wise deletion). Factors were compared between cases and controls using simple logistic regression and data were interpreted using Odds Ratios (OR) and 95% confidence intervals (95%CI). The statistical analysis was performed in STATA 16.1 and statistical significance was defined based on confidence intervals. The datasets used and analyzed during the current study are available from the corresponding author on reasonable request.

## Results

### Demographic findings

A total of 728 families were approached and all agreed to answer the questionnaire, 182 (25%) cases, and 546 (75%) controls. Caregivers (mainly mothers) share similar occupations, wages, socioeconomic status, places of residence, type of insurance, and education (Table 1). Patients among cases were older and had a higher median number of siblings than controls (Table 2). Having received antibiotics in the last 3 months increased the probability of self-medication, but if either parent had an education beyond high school the probability of self-medication was lower (Table 2).

**Table 1:** Association between antibiotic self-medication and demographic characteristics of the caregivers

**Characteristic**		**Cases**	**%**	**Controls**	**%**	**OR**	**CI 95%**
		***N=*182**		***N=*546**		**-**	**-**
Occupation	*Employee / Independent*	138	76	354	65	*(Ref)*	
	Unemployed	29	16	117	22	0.64	0.40 - 0.99
	Student	8	4	32	6	0.64	0.28- 1.40
	Employed	6	3	37	7	0.42	0.17 - 1.00
	Pensioned	1	1	3	1	0.85	0.08 - 8.20
Salary+	Less than a minimum wage	27	15	56	10	1.35	0.81- 2.25
	*1-3 times the minimum wage*	110	60	309	57	*(Ref)*	
	4 or more times the minimum wage	8	4	33	6	0.68	0.30- 1.50
	No income	37	20	148	27	0.70	0.46- 1.06
Socioeconomic Status^a^	*Level 1*	26	14	73	13	*(Ref)*	
	Level 2	95	52	253	46	1.04	0.63- 1.73
	Level 3	60	33	205	38	0.82	0.48- 1.39
	Level 4	1	1	13	2	0.31	0.05- 1.76
	Level 5	0	0	2	0	0.55	0.26- 11.93
Location of residence	Urban	175	96	523	96	1.09	0.46- 2.60
	*Rural*	7	4	23	4	*(Ref)*	
Healthcare contribution^b^	*Contributory regime*	140	77	417	76	*(Ref)*	
	Subsidized regime	41	23	123	23	0.99	0.66- 1.48
	Special regime	1	1	6	1	0.49	0.06- 4.16
Caregiver interviewed	*Mother*	138	77	444	83	(*Ref*)	
	Father	29	16	61	11	1.52	0.94 - 2.47
	Grandparent	7	4	16	3	1.41	0.57- 2.49
	Other^c^	6	3	13	22	1.48	0.55- 3.98
Mother’s schooling	*Up to and including high school*	112	62	259	47	(*Ref*)	
	**Higher**	**70**	**38**	**287**	**53**	**0.56**	**(0.40- 0.79)**
Father’s schooling	*Up to and including high school*	128	70	128	23	(*Ref*)	
	**Higher**	**54**	**30**	**325**	**60**	**0.62**	**(0.43- 0.89)**
Caregiver’s level of education^d^	*Up to and including high school*	11	73	26	63	*(Ref)*	
	Higher	4	27	15	37	0.63	(0.17- 2.33)

*Ref* Reference group for categorical logistic analysis OR and CI95% compared to reference category by simple logistic regression, *OR* Odds ratio, *CI 95%* 95% confidence interval. In italics are the reference categories for group comparisons and in bold are statistically significant categories

+ Minimum wage in Colombia is $287 dollars (with representative exchange rate of December 2021)

^a^Socioeconomic level: Based on an official strata division (1 through 6), greater number means higher income

^b^Healthcare system in Colombia uses a shared-contribution system, the contributory regime group contribute a percentage of their salary, the subsidized regime group do not have resources and are subsidized by the contributory regime group, and the special regime are those who belong to other contribution systems (military or teachers for example)

^c^Uncle/Aunt, Sibling, Stepmother, Cousin, Neighbor, Guardian

^d^Other than mother or father

In italics are the reference categories for group comparisons and in bold are statistically significant categories

**Table 2:** Association between antibiotic self-medication and demographic characteristics of children

**Characteristic**		**Case *N=*182**	**%**	**Control *N=*546**	**%**	**OR**	**CI 95%**
Sex (Female), n (%)		95	52	257	47	1.22	0.87- 1.72
**Age (years), median (IQR)**		**5**	**(8)**	**2**	**(5)**	**1.13**	**1.09- 1.17**
**Weight (Kg), median (IQR)**		**20**	**(21)**	**13**	**(11)**	**1.04**	**1.02- 1.05**
**Height (cm), median (IQR)**		**112**	**(47)**	**89**	**(42)**	**1.02**	**1.01- 1.03**
**Number of siblings, median (IQR)**		**1**	**(2)**	**1**	**(2)**	**1.25**	**1.09- 1.43**
Position among siblings^a^	*Oldest*	32	24	65	18	*(Ref)*	
	Youngest	101	76	289	82	0.71	0.44- 1.15
**Use of antibiotics in the last 3 months**		**108**	**59**	**103**	**19**	**6.27**	**4.35- 9.04**
Use of antibiotics by some sibling in the last 3 months^a^		28	21	66	19	1.16	0.71- 1.90
Infectious diagnosis^b^							
URTI: Common cold, Croup, sinusitis, tonsillitis)		75	41	161	29	*(Ref)*	
Ear infection		13	7	39	7	0.72	0.36- 1.42
**LRTI: Bronchiolitis, wheezing, pneumonia**		**13**	**7**	**64**	**12**	**0.43**	**0.23- 0.84**
**Gastrointestinal infection**		**33**	**18**	**178**	**33**	**0.39**	**0.25- 0.63**
Urinary tract infection		18	10	46	8	0.84	0.45- 1.55
Skin and soft tissues		13	7	30	5	0.93	0.45- 1.88
Eyes and mouth		13	7	21	4	1.33	0.63- 2.79
Systemic: Bacteremia, Osteoarticular, Dengue		4	2	7	1	1.22	0.34- 4.32

*IQR* Interquartile range, *Ref* Reference group for categorical logistic analysis OR and CI95% compared to reference category by simple logistic regression, *OR* Odds ratio, CI 95% 95% confidence interval, *URTI* Upper respiratory tract infections, *LRTI* Lower respiratory tract infections

^a^Data was calculated with the number of cases and controls that report having a sibling (Denominator for Cases *N=*133 and Controls *N=*354)

^b^Infection diagnosis as given by the ED provider. In italics are the reference categories for group comparisons and bold are statistically significant categories

The most frequent diagnosis amongst the cases was an upper respiratory tract infection (URTI), (41% of the cases (75/182)). Having a lower respiratory tract infection (LRTI) (OR of 0.43 [95% CI 0.23-0.84] or a gastrointestinal infection (OR of 0.39 [95% CI 0.25-0.63] was associated with less self-medication compared to URTI.

**Fig. 1:**
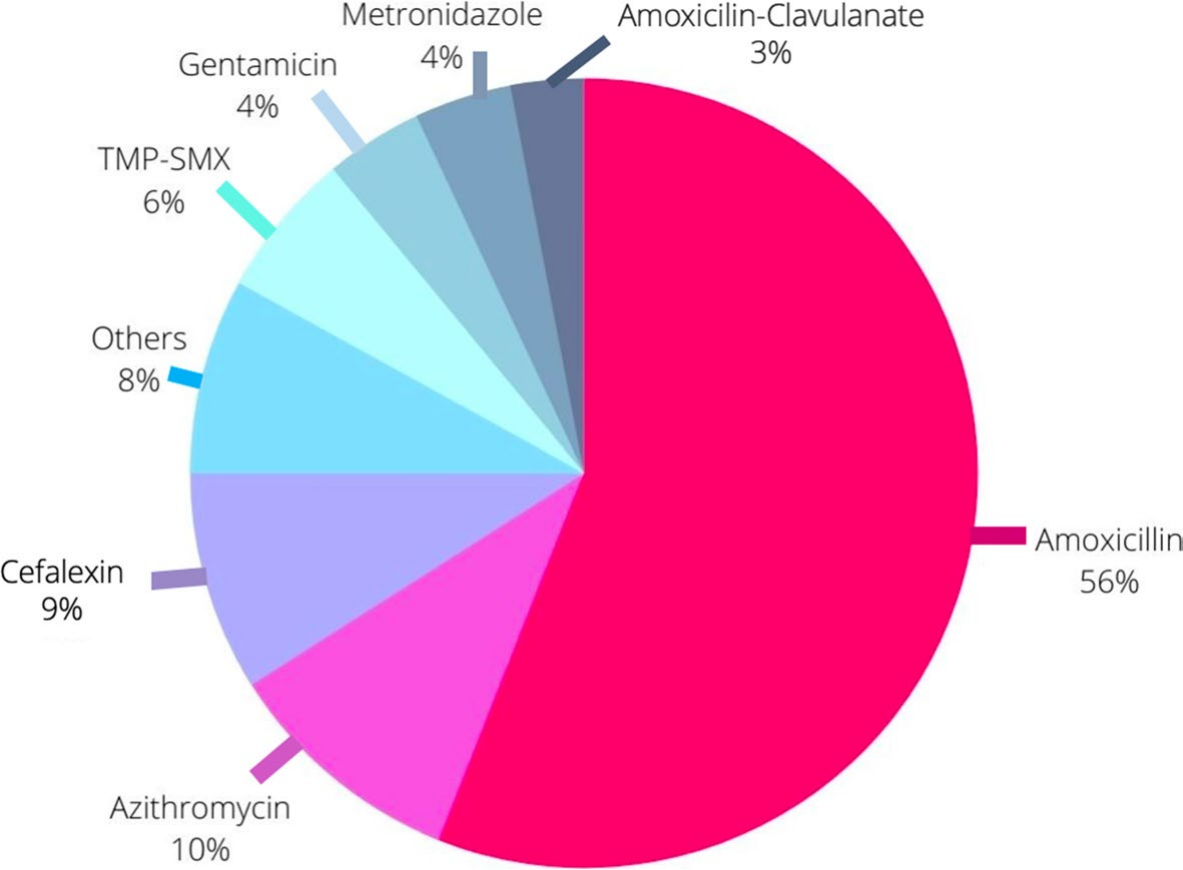
Type of antibiotic self medicating. TMP-SMX: Trimethoprim/sulfamethoxazole Others* Ampicillin, Ciprofloxacin, Clarithromycin, Clindamycin, Dicloxacillin, Nifuroxazide, Norfloxacin, Trimethoprim Sulfadiazine (1% each)

Amoxicillin was the most frequently self-medicated antibiotic for 108 patients (56%), followed by azithromycin and cephalexin (Fig. 1). Duration from symtoms onset to emergency room (ER) ranged from 2 to 6 days, and they took antibiotics in a range of 1 to 3 days prior to ER arrival.

### Caregiver source of the medication and reason for antibiotic self-administration

The antibiotic was recommended most frequently by pharmacy staff (55% of cases), followed by a family member (24%) and friends (1%) or neighbors (1%). Prior to starting the antibiotic, 55% of caregivers reported that they did not read the instructions for use. When asked about whether the initially commenced dose was altered in subsequent days, 75% reported that it remained unchanged. Among the symptoms that most frequently led to self-medication were fever (38%), odynophagia (17%), and gastrointestinal symptoms (12%). When asked for reasons why caregivers self-medicated the patients, 47% did so because they found improvement in previous visits and illness, 20% reported that the severity of symptoms required antibiotics, 14% found difficulties getting access to health care (lack of time, not having enough money to access to the health service, distance to the healthcare center, and other problems with the healthcare system) and 9% reported other reasons such as: no improvement with anti-inflammatory medications, advice from the pharmacy staff, belief in improvement of symptoms with antibiotics and a nurse in the family.

With respect to the different attitudes adopted by the caregiver (Table 3), cases that always asked doctors to prescribe antibiotics were more likely to practice self-medication (OR 3.92 [95% CI 1.59-9.66]). Similarly, those who said they bought antibiotics without a prescription – sometimes (OR 11.70 [95% CI 7.09-19.31]), frequently (OR 23.66 [95% CI 11.76-47.59]), or always (OR 36.81 [CI 95% 18.32-73.95]), and those who recommended that relatives use antibiotics (OR 2.9 [95% CI 1.75-4.82] were more likely to practice self-medication. Thirty-seven percent of caregivers that self-medicated always read the prescribing information in the medication package in comparison with only 19% of the controls (OR 3.83 [95% CI 2.57-5.71]).

**Table 3:** Attitudes of Caregivers Towards Self-medication

**Question**		**Cases**	**%**	**Controls**	**%**	**OR**	**CI 95%**
		***N=*182**		***N=*546**		**-**	**-**
When you go to a medical appointment, do you ask the doctor for antibiotics?	*Never*	*138*	*76*	*443*	*81*	*(Ref)*	
	Sometimes	27	15	84	15	1.03	0.64- 1.65
	Frequently	6	3	10	2	1.93	0.68- 5.39
	**Always**	**11**	**6**	**9**	**2**	**3.92**	**1.59- 9.66**
When you go to the drugstore, do you buy antibiotics without a medical prescription?	*Never*	*23*	*13*	*381*	*70*	*(Ref)*	
	**Sometimes**	**89**	**49**	**126**	**23**	**11.70**	**7.09 - 19.31**
	**Frequently**	**30**	**16**	**21**	**4**	**23.66**	**11.76 - 47.59**
	**Always**	**40**	**22**	**18**	**3**	**36.81**	**18.32- 73.95**
Are you and your family used to recommending antibiotics to your relatives?	*Never*	*81*	*45*	*303*	*55*	*(Ref)*	
	Sometimes	47	26	160	29	1.09	0.73- 1.65
	Frequently	19	10	38	7	1.87	1.02 - 3.42
	**Always**	**35**	**19**	**45**	**8**	**2.90**	**1.75- 4.82**
When you start taking an antibiotic, do you check the dose and indications online before using it?	*Never*	*111*	*61*	*306*	*56*	*(Ref)*	
	Sometimes	21	12	81	15	0.71	0.42- 1.21
	Frequently	10	5	34	6	0.81	0.38- 1.69
	Always	40	22	125	23	0.88	0.58- 1.39
When you start taking an antibiotic, do you check the recommendation insert before using it?	*Never*	*67*	*37*	*351*	*64*	*(Ref)*	
	**Sometimes**	**21**	**12**	**38**	**7**	**2.89**	**1.59- 5.24**
	**Frequently**	**20**	**11**	**56**	**10**	**1.87**	**1.05- 3.32**
	**Always**	**67**	**37**	**101**	**19**	**3.83**	**2.57- 5.71**
Do you usually keep antibiotics at home?	*Never*	*111*	*61*	*357*	*65*	*(Ref)*	
	Sometimes	42	23	128	23	1.05	0.70- 1.58
	Frequently	7	4	14	3	1.61	0.63- 4.08
	Always	22	12	47	9	1.51	0.87- 2.61

In italics are the reference categories for group comparisons and in bold are statistically significant categories

*OR* Odds ratio, *CI 95%* 95% confidence interval

### Caregiver knowledge

The knowledge caregivers had about the practice of self-medication is presented in Table 4. Of note, 29% of self-medicating caregivers believe that antibiotics are an effective treatment against the common cold in comparison with 22% in the control group (OR 1.55 [95% CI 1.03-2.35]). Also, 66% of caregiver’s who practiced self-medication believed that antibiotics shorten the duration of viral infections compared to 50% of controls (OR 2.29 [95% CI 1.43-3.66]). Forty-five percent of the cases believe that antibiotic therapy can be discontinued once the fever passes, in contrast to only 29% of the controls (OR 2.11 [95% CI 1.46-3.03]). In addition, only about one in ten cases and controls linked the use of antibiotics to the risk of generating bacterial resistance. However, approximately one half of the caregivers in both groups do associate antibiotic use with the onset of diarrhea and rash. Both groups, (67% of the controls and 70% of cases), agree that the level of self-medication is high in Colombia.

**Table 4:** Knowledge caregivers have about self-medication

**Question**	**Response**	**Cases**	**%**	**Controls**	**%**	**OR**	**CI 95%**
		***N=*182**		***N=*546**		**-**	**-**
Acetaminophen	***Does not work for infections***	124	68	397	73	***(Ref)***	
	It is better than an antibiotic	4	2	11	2	1.16	0.36- 3.72
	It works like an antibiotic	11	6	24	4	1.46	0.69- 3.08
	I don’t know	43	24	114	21	1.21	0.80- 1.81
Antibiotics	***Do not work for the common cold.***	74	41	265	49	***(Ref)***	
	Work the same as acetaminophen.	1	1	17	3	0.22	0.03- 1.61
	**Work for the common cold.**	**53**	**29**	**122**	**22**	**1.55**	**1.03- 2.35**
	I don’t know	54	30	142	26	1.36	0.91- 2.04
When an infection is caused by a virus, the ideal treatment is an antibiotic.	***False***	35	19	188	34	***(Ref)***	
	**True**	**97**	**53**	**225**	**41**	**2.31**	**1.50- 3.56**
	**I don’t know**	**50**	**27**	**133**	**24**	**2.01**	**1.24- 3.28**
When an infection is caused by a virus, an antibiotic will shorten the duration of the infection.	***False***	26	14	135	25	***(Ref)***	
	**True**	**121**	**66**	**271**	**50**	**2.29**	**1.43- 3.66**
	I don’t know	35	19	140	26	1.29	0.74- 2.26
Antibiotics are very safe drugs and do not cause any adverse effects.	***False***	84	46	278	51	***(Ref)***	
	True	35	19	25	23	0.93	0.59- 1.45
	I don’t know	63	35	143	26	1.45	0.99- 2.14
Antibiotics increase the risk of bacterial resistance.	***False***	85	47	162	48	***(Ref)***	
	True	14	8	56	10	0.77	0.41- 1.45
	I don’t know	83	46	228	42	1.12	0.79- 1.59
Antibiotics cause adverse effects such as diarrhea and rash.	***False***	88	48	317	58	***(Ref)***	
	True	26	14	74	14	0.78	0.47- 1.29
	I don’t know	68	37	155	28	1.24	0.73- 2.10
There are many new antibiotics for treating infections.	***False***	7	4	39	7	***(Ref)***	
	True	87	48	235	43	0.48	0.20- 1.12
	I don’t know	88	48	272	50	0.87	0.61- 1.23
Antibiotics should be used when one has a fever.	***False***	112	62	380	70	***(Ref)***	
	True	39	21	92	17	1.44	0.93- 2.21
	I don’t know	31	17	74	14	1.42	0.88- 2.27
In Colombia, the level of self-medication is low	***False***	122	67	383	70	***(Ref)***	
	True	30	16	78	14	1.21	0.75- 1.93
	I don’t know	30	16	85	16	1.10	0.69- 1.76
Antibiotics should be discontinued when the fever is gone.	***False***	78	43	319	58	***(Ref)***	
	**True**	**81**	**45**	**157**	**29**	**2.11**	**1.46- 3.03**
	I don’t know	23	13	70	13	1.36	0.80- 2.30

In italics are the reference categories for group comparisons and in bold are statistically significant categories

Only one response was accepted, caregivers were instructed to select the best single response

*OR* Odds ratio, *CI 95%* 95% confidence interval

## Discussion

This study identified the characteristics of children and their caregivers who self-medicate antibiotics, and explored caregivers’ attitudes and knowledge regarding appropriate antibiotic use. Suboptimal behaviors and limited knowledge of rational antibiotic use were strongly associated with antibiotic self-administration. In addition, there was a higher probability of antibiotic self-medication in cases with a greater number of siblings and use of antibiotics in the previous three months.

### Patient characteristics

In the present study, self-medicated children were older than those in the control groups (median age 5 years versus 2 years) and had a six-fold higher probability of having received antibiotics over the previous 3 months than the control group. This recent antibiotic use being associated with subsequent self-medication is similar to findings reported in Rio de Janeiro [17]. Upper respiratory infections were the main reason for self-medication in our study, which is consistent with international pediatric literature. The predominance of self-medication for conditions considered “non-serious illness” has previously been widely described, including in the study by Tuyishimire et al. [18]. In the current study, a significant increase in the risk of self-medication was found if there were multiple siblings in the family. It is possible that the mother’s experience gained by treating her children for previous pathologies or symptoms that have been satisfactorily resolved in the older children led to the practice of self-medication [19].

### Caregiver education status

The effect of the caregivers’ educational level on self-medication practices varies depending upon the population studied. In our study, we found that higher levels of caregiver education were associated with reduced self-medication. Likewise, studies in China and Mozambique describe a strong association between a high level of schooling and a lower tendency to self-medicate with antibiotics [7, 20–23]. In contrast, studies in Cameroon and Trinidad and Tobago show that the population with a higher educational level was more likely to self-medicate [23, 24], whilst Widayati et al. found no association between educational level and self-medication [25]. Other than educational status, several social, cultural and economic factors that may influence self-medication practices have been reported previously. These include: parents’ financial means; government policies that prohibit dispensing of antibiotics without a medical prescription [26]; pharmacies that doesn’t dispense exact quantities of the prescribed antibiotic [27–30]; difficulties accessing the health system due to waiting times and high cost [31]; and endemic local diseases that mean that at-risk populations are frequently using antibiotics [7, 32–36].

### Attitudes of caregivers with respect to self-medication

Caregivers who self-medicated, more frequently requested antibiotics at the doctor’s appointment and were more likely to purchase antibiotics from pharmacies without a prior medical prescription. These caregivers were also influenced by recommendations from a friend or relative or, more frequently, by the pharmacy staff [20, 35, 36]. In Colombia, pharmacies are commonly staffed by sales staff who do not have formal pharmaceutical training or registration. Other factors that are described as contributing to self medication that were not explored in our study are the erroneous belief in the effectiveness of antibiotics to relieve self-limiting disease, the perception of previous success and absence of previous complications [9, 14, 37] and using antibiotics as a self-care measure [38, 39].

In our study, caregivers who self-medicated were significantly more likely to check the package insert prior to medicating, than controls who did not self-medicate. Despite this, over half of cases in our study reported not reading the instructions prior to administration. A cross-sectional study in Cameroon found that the instructions on the use of antibiotics were seven times more likely to be read by those who bought antibiotics with a medical prescription, compared to those who bought them without a prescription [24]. Similarly, a study in Guatemala found that more than 80% of self-medicated individuals did not read the instructions about the use of antibiotics on the recommendations insert [40].

### Knowledge caregivers have about self-medication

When the beliefs or knowledge gaps that could explain the self-medication behavior were analyzed, our study found that the difference between a bacterial or viral infection is not clearly understood and that antibiotics are considered an effective treatment against viral infections and a useful strategy for managing the common cold. This correlates with the information presented in literature [38, 39]. Navaro, et al, found that illnesses considered mild and the common cold were the most frequent reasons for using antibiotics without prior medical advice [41]. The higher the level of knowledge regarding antibiotics, the lower the probability of self-medicating or using antibiotics as a treatment against viral infections [42]. In Cameroon close to 87% of the respondents did not know the target of antibiotics, believing that it could be used as a treatment against all kinds of microorganisms and a large percentage of them believed antibiotics could be used to treat viral infections [24]. In our study, cases considered antibiotics to be effective against viral infections and did not know their appropriate use in case of fever. Similar erroneous perceptions were identified in other countries [7, 25, 43–45].

In general, there is a significant lack of awareness of the potential adverse effects of indiscriminate use of antibiotics. Our study found that approximately one fifth of caregivers (19% for cases and 23% for controls) believe that antibiotics are safe medications and do not have adverse effects. Other studies have shown that mothers who have better knowledge about the use and risks of antibiotics are less likely to administer them to their children without a prior prescription [15, 46].

Self-medication with antibiotics is multifactorial, and future studies should address the factors found here, including education about the misconceptions that motivate this behavior. Educational interventions regarding the principles of antimicrobial stewardship should target pharmacy staff and people who attend pharmacies. One action that could be performed in the future to limit the problem of self medication of antibiotics is a public policy that regulates the access to antibiotics only with a medical prescription. Also giving the exact dose as the prescription indicates could limit the possibility of storing antibiotics at home,

This study has some limitations, for example, it only included a part of the population in Colombia with health insurance in lower-middle income, which does not represent the entire population of the country or of other countries. Another limitation was that although patients could be recruited every day as all staff was trained and reminded each month, study members were not present 24 hours a day, therefore some cases may not have been included and this may generate a selection bias. In addition, the recruitment phase was interrupted by the COVID-19 pandemic and the mandatory quarantine in Colombia drastically reduced the consultation of the pediatric population for emergency care, so recruitment prior to the target sample size was interrupted, obtaining a lower power (75%) with the final population size.

## Conclusion

There is a greater chance of self-medicating with antibiotics with each additional sibling, amongst older children, and when one has used them in the previous 3 months. In contrast, the probability declines significantly with caregivers who have a higher educational level. Knowledge of the instructions for antibiotics is deficient in this population, and among the attitudes most likely to lead to self-medication were actively requesting antibiotics from a doctor, acquiring them without a prescription and on the recommendation of a relative.

## Data Availability

The datasets used and analyzed during the current study available from the corresponding author on reasonable request.
